# The RUFYs, a Family of Effector Proteins Involved in Intracellular Trafficking and Cytoskeleton Dynamics

**DOI:** 10.3389/fcell.2020.00779

**Published:** 2020-08-11

**Authors:** Rémy Char, Philippe Pierre

**Affiliations:** ^1^Aix Marseille Université, Centre National de la Recherche Scientifique, Institut National de la Santé et de la Recherche Médicale, Centre d’Immunologie de Marseille-Luminy, Marseille, France; ^2^Institute for Research in Biomedicine and Ilidio Pinho Foundation, Department of Medical Sciences, University of Aveiro, Aveiro, Portugal; ^3^Shanghai Institute of Immunology, School of Medicine, Shanghai Jiao Tong University, Shanghai, China

**Keywords:** RUFY, cancer, neurodegenerative diseases, immunity, RUN, FYVE, phosphatidylinositol 3-phosphate, cytoskeleton

## Abstract

Intracellular trafficking is essential for cell structure and function. In order to perform key tasks such as phagocytosis, secretion or migration, cells must coordinate their intracellular trafficking, and cytoskeleton dynamics. This relies on certain classes of proteins endowed with specialized and conserved domains that bridge membranes with effector proteins. Of particular interest are proteins capable of interacting with membrane subdomains enriched in specific phosphatidylinositol lipids, tightly regulated by various kinases and phosphatases. Here, we focus on the poorly studied RUFY family of adaptor proteins, characterized by a RUN domain, which interacts with small GTP-binding proteins, and a FYVE domain, involved in the recognition of phosphatidylinositol 3-phosphate. We report recent findings on this protein family that regulates endosomal trafficking, cell migration and upon dysfunction, can lead to severe pathology at the organismal level.

## Introduction

The organization of cells into multiple membranous compartments with specific biochemical functions requires complex intracellular traffic and sorting of lipids and proteins, to transport them from their sites of synthesis to their functional destination. Intracellular transport involves lipid vesicles or tubules with the capacity to fuse with one another or to be secreted. They collectively participate in the dynamic exchanges necessary for cell homeostasis (Rothman, 2002; Søreng et al., 2018). Membrane traffic is tightly coordinated with protein synthesis, signal transduction of environmental stimuli and cytoskeleton organization, allowing the implementation of key cellular functions such as endocytosis, exocytosis, or migration (McMahon and Gallop, 2005; Habtezion et al., 2016; Vega-Cabrera and Pardo-López, 2017; MacGillavry and Hoogenraad, 2018; Margaria et al., 2019; Tapia et al., 2019; Buratta et al., 2020; Stalder and Gershlick, 2020).

Several families of molecular components required for orchestrating membrane vesicle exchange and transport during this process are conserved. They include adaptor and coat proteins, small GTP-binding proteins (GTPases), as well as Synaptosome Associated Protein (SNAP) Receptor (SNARE) proteins and SNARE binding proteins (Juliano, 2018). The vast superfamily of GTPases is involved in the establishment or regulation of virtually every step of intracellular membrane trafficking. They behave as molecular switches that can alternate between active and inactive states, through GTP binding and hydrolysis into GDP (Takai et al., 2001; Stenmark, 2009). The largest group of GTPases involved in intracellular membrane traffic is the Rab proteins family (Lamb et al., 2016). Rab GTPases specifically localize to different intracellular compartments, regulating vesicle formation and sorting, as well as transport along the cytoskeletal network. Each Rab protein can be recruited to specific membrane subdomains of a defined organelle and is associated to multiple effectors controlling membrane fusion and trafficking. Rab interaction with the membrane fusion complexes and cytoskeleton regulators is therefore crucial for cellular functions, including endocytosis and autophagy (Chen and Wandinger-Ness, 2001; Bruce et al., 2010; Geng et al., 2010; Thomas and Fromme, 2020; Yuan and Song, 2020).

**FIGURE 1:**
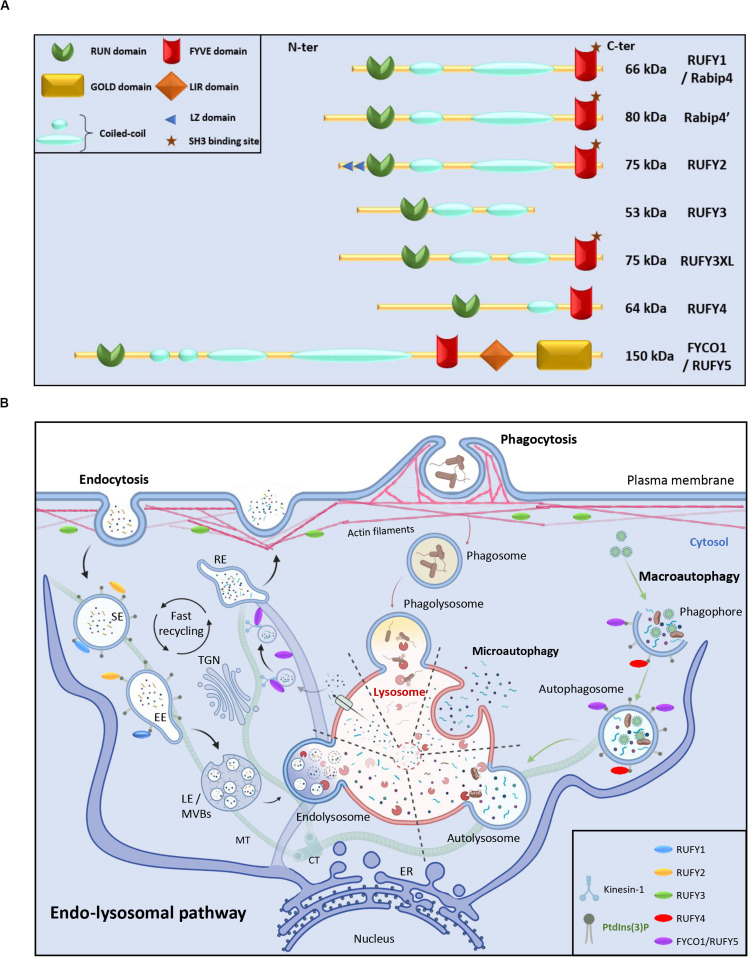
RUN and FYVE domain containing-proteins in the endo-lysosomal pathway. **(A)** Schematic representation of the RUFY proteins family. **(B)** Description of the endo-lysosomal and autophagy pathways and presumed functional locations of RUFY proteins. Extracellular material is ingested by endocytosis or phagocytosis. The action of different endosomes allows cargo to be sorted, recycled or degraded in a complex and regulated process involving fusion, maturation and transport along the cytoskeleton. Alternatively, during autophagy, obsolete components present in cytosol are captured in autophagosomes prior fusion with lysosomes and degradation (macroautophagy) or directly internalized through endosomal invagination (microautophagy). SE, sorting endosome; EE, early endosome; TGN, trans golgi network; LE, late endosome; MVBs, multi vesicular bodies; RE, recycling endosome, MT, microtubule; CT, centrioles; ER, endoplasmic reticulum. The location of PI3P and RUFY proteins known activity is shown. Created with BIoRender.com.

Here, we review the literature concerning a less-well known family of proteins involved in the complex biochemical crosstalk established between the cytoskeleton and intracellular vesicles. This small group of proteins was named RUFY for “RUN and FYVE domain-containing.” RUFYs share a common structural domain organization, including an N-terminal RUN domain, one or several coiled-coil (CC) repeats and a C-terminal FYVE domain (Figure 1A). The molecular structures of the different RUFY proteins has been described (Dunkelberg and Gutierrez-Hartmann, 2001; Mari et al., 2001; Kukimoto-Niino et al., 2006; Kitagishi and Matsuda, 2013), but their function in endocytic regulation and their physiological relevance at the organismal level are still poorly characterized (Kitagishi and Matsuda, 2013; Terawaki et al., 2016). We revisit here how the *rufy* gene family was annotated, and propose the addition of a novel member, the *fyco1* (FYVE and Coiled-coil containing domain 1) gene given its sequence and functional similarities with the other *rufy* genes (Pankiv et al., 2010; Terawaki et al., 2015). We also highlight recent findings on the implication of RUFY proteins in the regulation of cytoskeleton and endosome dynamics and their contribution to immunity, cancer and neurodegenerative diseases.

### Endocytosis and Autophagy

Endocytosis and autophagy are membrane traffic pathways required for degradation and recycling of extracellular and intracellular components, respectively (Birgisdottir and Johansen, 2020). These pathways have a common endpoint at the lysosome, where their cargo is degraded. These both pathways intersect at several stages throughout vesicle formation, transport and fusion and share some of the components of their molecular machineries (Figure 1B).

There are numerous co-existing endocytic pathways, which initiate by the formation of nascent endocytic vesicles formed from plasma membrane invaginations and scissions. These endocytic vesicles undergo homotypic fusion and are rapidly targeted to sorting endosomes (SE). Sorting events initiated in SE determine the fate of internalized cargo molecules, such as recycling to plasma membrane, degradation in lysosomes, or other trafficking events (Naslavsky and Caplan, 2018; Figure 1B). On their way to degradation, sorted cargo accumulate in early endosomes (EE), that further mature into late endosomes (LE) through multiple events of cargo and lipid sorting. Late endosomes adopt a membrane organization termed multivesicular bodies, that are enriched in lysobisphosphatidic acid and contain intraluminal vesicles (Gruenberg, 2020). Next, LE potentiate their hydrolytic competence by fusing with lysosomes (Pillay et al., 2002) resulting in the degradation of their contents, providing nutrients and key factors to the cell (Doherty and McMahon, 2009; Kaksonen and Roux, 2018). Notably, endosomes play a role in signal transduction by serving as signaling platforms either for surface activated receptors like Toll-like receptors and epidermal growth factor receptor or metabolic sensors such as mechanistic target of rapamycin complex 1 (mTORC1; Argüello et al., 2016). Often they promote the degradation of their targets, leading to signal termination (Chung et al., 2010). The endocytic pathway has also specialized functions in differentiated cells such as neurotransmitter release and recycling in neurons, or antigen processing and presentation in professional antigen presenting cells, like B cells or dendritic cells (Argüello et al., 2016; Solé-Domènech et al., 2016; Hinze and Boucrot, 2018). Endocytosis events and endosomes positioning is highly dependent on the dynamic and spatial re-organization of the different cytoskeleton networks that include actin, intermediate filaments, or microtubules (Fletcher and Mullins, 2010; Pegoraro et al., 2017).

Complementary to endocytosis, autophagy is an intracellular process by which cells degrade and recycle their own cytoplasmic materials (Mizushima and Komatsu, 2011). Autophagy plays a central role in many physiological processes including stress management, development, immunity and aging (Puleston and Simon, 2014; Zhong et al., 2016; Fîlfan et al., 2017; Moretti et al., 2017; Doherty and Baehrecke, 2018). Autophagy is partially controlled though mTORC1 activity and is responsible for degradation and recycling of misfolded proteins, as well as obsolete organelles (Galluzzi et al., 2017). The endpoint of autophagy is to deliver cytoplasmic material to lysosomes, where like for endocytosed cargo, it is degraded. Several autophagy processes can be distinguished based on the entry mode of the cytosolic components destined for degradation (Figure 1B). Macroautophagy involves engulfment of cytoplasmic contents into a double membrane vesicle termed the autophagosome. The autophagosome fuses then with lysosomes, becoming an autolysosome, in which its cargo is degraded (Galluzzi et al., 2017). The presence of specific phosphoinositides lipids, together with Rab GTPases, at a given membrane compartment is often directly correlated with compartment function. One of the common mechanism regulating endocytosis and autophagy is an accumulation of phosphatidylinositol 3-phosphate (PtdIns(3)P) at surface of EE and on intraluminal vesicles of multivesicular endosomes and on autophagosomes (Nascimbeni et al., 2017; Figure 1B). PtdIns(3)P is also observed at sites of LC3−associated phagocytosis another pathway of internalization used by the cells to ingest large particulate material or microbes. PtdIns(3)P is therefore a beacon used by the cellular machinery to regulate endosomal sorting and autophagy (Birgisdottir and Johansen, 2020).

### RUN Domains

**FIGURE 2:**
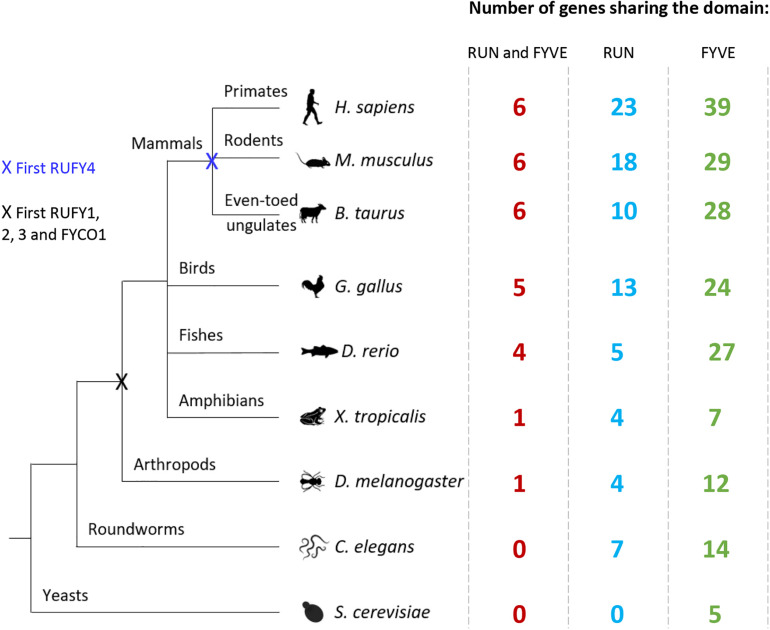
Evolution of RUN and FYVE domain or *rufy* genes among living organisms. Diagram illustrating the evolution of the *rufy* genes. Species representative of various taxonomic groups are listed, data were extracted from the Differential Expression Atlas Genes database (EMBL-EBI). Next to each species studied, the number corresponds to the number of genes having in its sequence a FYVE (green), RUN (blue) or both (red) domain. The “X” corresponds to the appearance of a common *rufy* ancestor gene.

**FIGURE 3:**
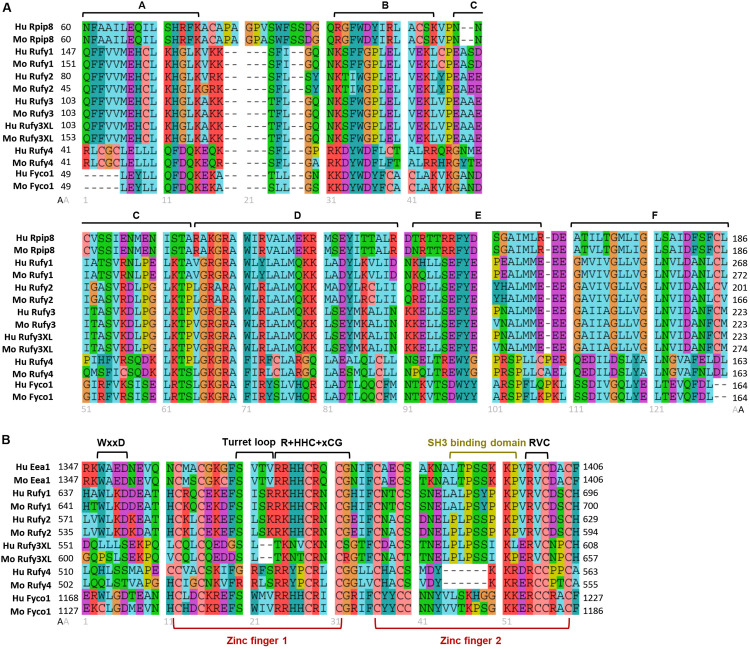
Molecular organization of RUN and FYVE domains from the RUFY proteins family. Alignment of the protein sequences of the RUN **(A)** and FYVE **(B)** domains of the RUFY proteins family in human and mouse. **(A)** RUN consensus blocks are represented by segments **(A–F)**. Rpip8 sequence is used as RUN domain reference, **(B)** FYVE conserved motives and zinc fingers are represented by segments. In the alignment, “x” is any amino acid and “+” represents positively charged amino acid. Eea1 sequence is used as FYVE domain reference. For all alignment, amino acids are colored according to their properties: Cyan for hydrophobic positions (A,V,I,L,M), turquoise for aromatic positions (F,Y,W,H), red for basic residues (K,R), purple for acidic residues (D,E), green for polar uncharged (N,Q,S,T), salmon for cysteine (C), orange for glycine (G) and yellow for proline (P). Gray numbers below alignment means the amino acids position after alignment. Black numbers surrounding the alignments represent the start (left) and end (right) positions of the domains in the peptide sequence of each protein. Alignment were realized with Seaviewer analyzer software (Gouy et al., 2010). Accession numbers for protein are following: human Rpip8 (NP_001138297.1), mouse Rpip8 (NP_058039.1), human Eea1 (NP_003557.3), mouse Eea1 (NP_001001932.1), human RUFY1 (NP_079434.3), mouse RUFY1 (NP_766145.1), human RUFY2 (NP_060457.4), mouse RUFY2 (NP_081701.2), human RUFY3 (NP_055776.1), mouse RUFY3 (NP_081806.1) human RUFY3XL (NP_001032519.1), mouse RUFY3XL (NP_001276703.1), human RUFY4 (NP_940885.2), mouse RUFY4 (NP_001164112.1), human FYCO1 (NP_078789.2), mouse FYCO1 (NP_001103723.2).

**FIGURE 4:**
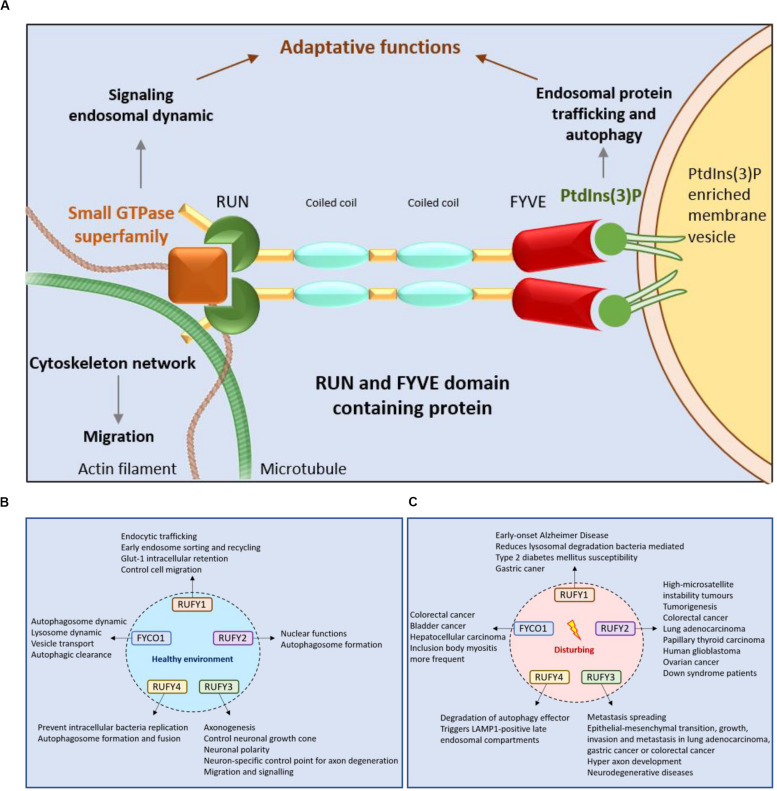
RUFY proteins are important for intracellular trafficking, signaling and cytoskeleton dynamics. **(A)** Schematic representation of the RUN and FYVE domains activity of RUFY proteins. RUN domains act on signaling, endosomal protein trafficking and cytoskeletal network dynamics via small GTPase proteins. FYVE domains bind PtdIns(3)P and regulates autophagy and endosome trafficking. **(B)** Function of RUFY proteins in homeostatic conditions. **(C)** Consequences of alterations in RUFY proteins functions at the cellular and organismal level.

The presence of a single copy of a RUN and a FYVE domain at their extremities is the key characteristic defining the RUFY family members. RUN domains were named after three proteins bearing similar peptide motifs, RPIP8, UNC-14 and NESCA (new molecule containing SH3 at the carboxy−terminus) (Ogura et al., 1997; Matsuda et al., 2000). RUN domains are present in multiple proteins (RUN proteins) in a large panel of organisms (Figure 2) and principally allow direct interactions with small GTPases of the Rap and Rab families (Callebaut et al., 2001; Yoshida et al., 2011). RUN domains adopt a hydrophobic globular structure bearing six conserved blocks named A to F (Figure 3A). These blocks correspond to eight α-helices and some 3_10_-helices. The first helix is crucial to limit hydrophobic exposure and maintain protein solubility of RUN-containing proteins (Callebaut et al., 2001; Kukimoto-Niino et al., 2006). In spite of strong conservation among the domains present in RUN-containing proteins, the proteins they interact with, their effectors, are highly variable (Mari et al., 2001) and the structural features of the RUN domain alone are not sufficient to define binding specificity for one or several members of the GTPase superfamily (Fukuda et al., 2011). Most RUN domain-bearing proteins bind small GTPases, but interactions with other molecules like kinesin 1 have also been described (Boucrot et al., 2005). A direct physical link between RUN proteins with actin filaments and microtubules has been also demonstrated (Torti et al., 1999), reinforcing the idea that these molecules are also critical for cellular functions requiring actin remodeling, such as migration or phagocytosis (Price and Bos, 2004; Bos, 2005; Miertzschke et al., 2007; Xu et al., 2007; Figure 4A). Additional functions for RUN domains have been described, for example for the RUN domain present in NESCA, which blocks TRAF6-mediated polyubiquitination of the NF-kappa-B essential modulator and consequently induces NF-kB activation. This is just one of the ways in which RUN proteins can act in signal transduction and the coordination of membrane traffic with actin dynamics upon external stimulation (Yoshida et al., 2011). As well as promoting endosomal fusion through their binding to Rab or Rap GTPases (Callebaut et al., 2001; Yoshida et al., 2011), their interaction with motor proteins, like kinesin or myosin, suggests a role for RUN domains in regulating vesicular and organelle transport (Callebaut et al., 2001; Yoshida et al., 2011). Via these different mechanisms, RUN proteins have been implicated in neuronal development (Honda et al., 2017b), signaling (Sun et al., 2012), migration (Yoshida et al., 2011), and regulation of various cellular function like endocytosis or exocytosis (Kitagishi and Matsuda, 2013).

### FYVE Domains

FYVE-domain-bearing proteins (for Fab1, YOTB/ZK632.12, Vac1, and EEA1) are specifically found in association with membranous organelles enriched in PtdIns(3)P and highly conserved among eukaryotes, including yeast (Hayakawa et al., 2007; Figure 2). FYVE domains adopt a zinc finger conformation (Misra and Hurley, 1999; Kutateladze and Overduin, 2001). In addition to FYVE, ten types of zinc finger folds have been characterized, including conventional, Gal4, GATA-1, TFIIS, MetRS, LIM, RING domain, PKC CRD, and PHD domains. Zinc fingers are structural conformations adopted by peptide chains upon coordination of two Zn^2+^ cations within a cysteine rich region (Schwabe and Klug, 1994; Stenmark et al., 1996). Unlike most molecules bearing zinc fingers, FYVE proteins display only one copy of the domain located at any position along the peptide chain, highlighting its autonomy as a structural unit. FYVE zinc fingers can stabilize protein-protein or protein-DNA/RNA interactions (Dunkelberg and Gutierrez-Hartmann, 2001). A “classical” FYVE domain has eight potential zinc coordinating tandem cysteine positions and is characterized by having basic amino acids around the cysteines. Many members of this family also include two histidine residues in a sequence motif including WxxD, CxxC, R+HHC+xCG and RVC where “x” means any amino acid and “+” a positively charged amino acid (Figure 3B). Most deviations from this sequence can reduce the domain affinity for zinc and destabilize it (Stenmark et al., 1996; Misra and Hurley, 1999; Stenmark and Aasland, 1999; Kutateladze and Overduin, 2001). Within this structural framework, specific modifications in the non-conserved residues of the domain can radically affect FYVE protein subcellular localization and function, by forming a “turret loop” and a dimerization interface (Hayakawa et al., 2004).

With regard to their affinity for PtdIns(3)P, FYVE domain-containing proteins are mostly found associated to EE or phagosomes (Stenmark et al., 1996; Gaullier et al., 1998; Stenmark and Aasland, 1999; Figure 4A). The presence of FYVE domains is therefore correlated to the regulation of membrane traffic, through specific recognition of PtdIns(3)P domains by “R+HHC+xCG” motifs (Gaullier et al., 1998), and modulation by associated phosphatidylinositol kinases. PtdIns(3)P is generated from phosphatidylinositol by Class III PtdIns 3-kinases (PI3K), like Vps34, on target membranes such as nascent autophagosome (omegasomes) (Melia et al., 2020), or EE (Di Paolo and De Camilli, 2006; Raiborg et al., 2013; Scott et al., 2014; Figure 1B). In turn, accumulation of PtdIns(3)P recruits and activates effector proteins containing FYVE domains, favoring transport or fusion of target organelles (Stenmark and Gillooly, 2001; Axe et al., 2008; Burman and Ktistakis, 2010; Schink et al., 2013). Affinity for PtdIns(3)P is determined by the pair of histidine residues present in the “R+HHC+XCG” motif of the FYVE domain (Stahelin et al., 2002; Diraviyam et al., 2003; Lee et al., 2005; He et al., 2009). This affinity can also be harnessed by FYVE proteins to link endosomes with mRNA, ribonucleoprotein particles (mRNP) and associated ribosomes, playing a role in their long-distance transport in the cell (Pohlmann et al., 2015). Importantly, many FYVE proteins homodimerize. Dimerization multiplies the conserved residues displayed by the different signature motifs present in the FYVE domain and contributes to a network of hydrogen bonding and electrostatic interactions that provides positive selection for binding several PtdIns(3)P head groups. PH-dependent insertion of FYVE domain into cell membranes (He et al., 2009; Pankiv et al., 2010) is reinforced by additional hydrophobic membrane interactions with the turret loop and/or tandem lysine residues. These non-specific interactions promote FYVE domain access to phosphate head groups, that are hindered by the close packing of lipid molecules. This bivalent mechanism increases therefore greatly FYVE domains specificity for PtdIns(3)P-enriched domains and discrimination against other mono- or polyphosphorylated PtdIns species (Misra and Hurley, 1999; Stenmark and Aasland, 1999; Dumas et al., 2001; Kutateladze and Overduin, 2001).

FYVE proteins are therefore key players in endocytosis and autophagy and mutations in FYVE domains can alter profoundly these functions, as well as cellular homeostasis (Kamentseva et al., 2020). For example, EEA1 protein (early endosome antigen 1) is known to be crucial for endosome dynamics and any mutation in its conserved residues or the oligomerization site can drastically reduce the affinity between its FYVE domain and PtdIns(3)P (Stenmark et al., 1996; Gaullier et al., 2000). In this context, RUFYs proteins, by bearing a N-terminal RUN domain, one or several copies of a coiled-coil domain next to a C-terminal FYVE domain (Figure 1A) have all the features required to carry-out specific adaptor functions to regulate endocytosis or autophagy by impacting on organelle fusion and mobility along the cytoskeleton.

### The RUFY Proteins Family

The RUFY family encompass four genes named *rufy1* to *4*, sharing homologies and displaying specific tissue expression and alternative splicing. *Rufy* genes are relatively conserved genes, absent from prokaryotes and fungi. Upon evolution, the emergence of the common ancestor appeared in vertebrates and arthropods, which possess one ortholog (*CG31064*) (Figure 2). No RUFY protein could be detected In *Caenorhabditis elegans* and only a FYVE-bearing protein (*T10G3.5)* considered as an ortholog of human EEA1 shows some sequence similarities with the RUFY family. *T10G3.5* exhibits PtdIns(3)P binding activity and is involved in endocytosis, being mostly expressed in epidermis and intestine of *C. elegans* (Hayakawa et al., 2007). In chordates, Rubicon (RUN domain and cysteine-rich domain containing, Beclin 1-interacting protein) and FYVE And Coiled-Coil Domain Autophagy Adaptor 1 (FYCO1), display structural and functional features, potentially categorizing them as RUFY proteins. Rubicon was identified as a component of the Class III PI3K complex and a negative regulator of autophagy and endosomal trafficking (Matsunaga et al., 2009; Zhong et al., 2009). Like RUFYs, Rubicon contains multiple functional domains that interact with other proteins, including a RUN, a CC and a FYVE-like domains (Wong et al., 2018). However, despite these similarities, the poor degree of sequence homology and the lack of conservation of its FYVE-like domain, which was found not to bind to PI(3)P (Burman and Ktistakis, 2010), prevented Rubicon’s integration within the RUFY proteins family, conversely to FYCO1, which we propose here to name RUFY5 and detail the characteristics below.

## RUFY1

RUFY1, previously named Rabip4 is an 80 kDa protein, mainly expressed in the brain, kidney, lung, placenta and testis. There are two RUFY1 isoforms Rabip4, and Rabip4’ that has an additional 108 amino acid upstream of the N-terminal RUN domain (Figure 1A). They were both shown to interact with the small endosomal GTPases Rab4, Rab5, and Rab14 (Fouraux et al., 2004; Vukmirica et al., 2006; Table 1). RUFY1 inactivation inhibits efficient recycling of endocytosed transferrin, implicating RUFY1 in the regulation of EE functions through cooperative interactions with Rab4 and Rab14 (Cormont et al., 2001; Yamamoto et al., 2010; Nag et al., 2018). This was further demonstrated by the alteration of epidermal growth factor receptor endocytic trafficking kinetics in cells depleted of RUFY1 (Gosney et al., 2018) and the hijacking of RUFY1 by the bacteria *P. gingivalis* to escape lysosomal degradation (Takeuchi et al., 2016). In melanocytes, RUFY1 was found to form a complex with rabenosyn-5, KIF3A-B, Rab4A and adaptor protein-3 (AP-3) to differentially regulate tyrosinase-related protein-1 and tyrosinase sorting in endosomes, contributing to melanosome maturation (Nag et al., 2018; Table 1). Moreover, silencing the Rabip4’ isoform of RUFY1 was shown to promote outgrowth of plasma membrane protrusions, and to regulate the spatial distribution of lysosomes at their tips, through an interaction with AP-3 (Ivan et al., 2012; Figures 1B, 4B). RUFY1 is also capable of controlling cell migration by regulating integrin trafficking (Vukmirica et al., 2006), presumably via endocytosis. In full agreement with a role of RUFY1 in regulating endosomal dynamics, a single nucleotide polymorphism (S705A) in the *rufy1* gene was associated with high blood glucose levels and type 2 diabetes mellitus susceptibility in an exome-wide association study (EWAS; Yamada et al., 2017). This result is consistent with the early finding that Rabip4 expression leads to Glucose transporter-1 (Glut-1) intracellular retention (Cormont et al., 2001). Interestingly RUFY1 display a SH3-binding motif “PxxPxP” embedded in the FYVE domain and is able to interacting with the epithelial and endothelial tyrosine kinase (ETK), and possibly regulates endocytosis through this interaction (Yang et al., 2002). Another EWAS, aiming to find early-onset Alzheimer’s Disease (AD) susceptibility genes, identified RUFY1 among genes involved in endo-lysosomal transport and known to be important for the development of AD (Kunkle et al., 2017; Figure 4C).

**TABLE 1:** Summary of RUFY proteins functional interactions.

Protein (Aliases)	Binding partner	Functions	Study
**RUFY1** (Rabip4; Rabip4’; ZFYVE12)	Rab4	Recycling endosomal trafficking	Cormont et al., 2001
	Etk	Regulation of endocytosis through its interaction with RUFY1	Yang et al., 2002
	Rab14	RUFY1’s recruitment, endosome tethering and fusion	Yamamoto et al., 2010
	AP-3	Regulates spatial distribution of lysosome	Ivan et al., 2012
	Rabenosyn-5/KIF3A- B/Rab4A/AP-3 complex	Sorting endosome pathway in endosomal membrane in melanocytes and segregates tyrosinase-related protein-1	Nag et al., 2018
	PODXL1	Increases cell proliferation, migration and invasion	Zhi et al., 2019
**RUFY2** (LZ-FYVE; Rabip4r; KIAA1537; FYVE13)	Rab33A/Rab4A/Rab6A	Endosome dynamic, Golgi complex-associated Rab33 and autophagosome formation on omegasomes	Fukuda et al., 2011; Kitagishi and Matsuda, 2013
	RET	Lead to a fusion of the RET tyrosine kinase domain to a RUN domain and a coiled-coil domain appear to be critical for tumorigenesis	Staubitz et al., 2019
**RUFY3** (Singar-1; RIPX; ZFYVE30; KIAA087)	Rap2	Control neuronal polarity	Janoueix-Lerosey et al., 1998
	Fascin	Control the growth of axons and neuronal growth cone	Wei et al., 2014
	Rab5/Rab33A	Acts on endosomal trafficking	Yoshida et al., 2010; Fukuda et al., 2011
	GPM6a-Rap2-STEF/Yial2 complex	Facilitates cell polarity	Honda et al., 2017a
	PAK1	Induce cell migration and invasion in gastric cancer	Wang et al., 2015
	FOXK1	Increases cells migration RUFY3-mediated with metastasis invasion in colorectal cancer	Xie et al., 2017a
	HOXD9	HOXD9 transactivate RUFY3 and it overexpression induce gastric cancer progression, proliferation and lung metastasis	Zhu et al., 2019
**RUFY4** (ZFYVE31)	Rab7	Autophagosome formation and lysosome clustering	Terawaki et al., 2015
**FYCO1/RUFY5** (ZFYVE7; RUFY3; CTRCT18; CATC2)	MAP1LC3A/B	Autophagosome formation and elongation	Cheng et al., 2016; Olsvik et al., 2015
	Rab7	Endosomal transport by acting with microtubule plus end-direction transport	Wang et al., 2011
	Kinesin-1	Allows translocation from the late endosome, lysosome and autophagosome to the plasma membrane through plus-end microtubule transport	Krauß and Haucke, 2015; Raiborg et al., 2016, 2015

## RUFY2

RUFY2 (or Leucine zipper FYVE-finger protein, LZ-FYVE) is a 75 kDa protein originally identified as an activating transcription factor-2 interactor embryogenesis (Dunkelberg and Gutierrez-Hartmann, 2001), preferentially located in the nucleus and expressed during. After development, RUFY2 expression remains high in the brain, lung, liver and the gastrointestinal tract (Yang et al., 2002). RUFY2 displays two N-terminal leucine zipper domains as well as a C-terminal FYVE-finger domain. Although it is likely to have a nuclear function at early stages of embryonic development, the presence of a FYVE domain suggests a cytoplasmic role for RUFY2 in regulating membrane traffic in fully differentiated cells. Importantly, the RUN domain of RUFY2 was shown to associate specifically with the Golgi complex-associated Rab33A (Fukuda et al., 2011; Table 1). Given the reported interaction of Rab33A and Rab33B with Atg16L and its putative role in regulating autophagy (Fukuda and Itoh, 2008), RUFY2 could contribute to autophagosome formation through a dual interaction with Rab33A and PtdIns(3)P on omegasomes (Figures 1B, 4B). Irrespective of its function, *rufy2* expression is subject to modulation by the micro RNA miR-155 (Bofill-De Ros et al., 2015), which is an important regulator of immune cells development and inflammatory responses (Ceppi et al., 2009). The *rufy2* gene is also frequently found mutated in cancer cells, with the most frequent mutations converting it into a strong target for nonsense mediated mRNA decay, thereby decreasing considerably its expression (Shin et al., 2011; Figure 4C).

## RUFY3

RUFY3, also known as Rap2-interacting protein X (RIPX) (Kukimoto-Niino et al., 2006) or Single Axon-Related 1 (Singar1) (Mori et al., 2007), is the best characterized member of the RUFY family. RUFY3, the smallest of the RUFY proteins with a molecular weight of 53 kDa (Figure 1A), is mostly expressed in neurons (Kitagishi and Matsuda, 2013). Neuronal RUFY3 is atypical, since it lacks a FYVE domain and is considered as part of the RUFY family based on strong sequence similarities with the other members, notably in the RUN and coiled-coil domains (Figure 2A). RUFY3 is distributed between the cytosol and at the plasma membrane, but not in intracellular vesicles, presumably because it lacks a FYVE domain. In artificial conditions, like following expression of the dominant gain of function mutant form of Rab5 (Q79L) in U937 cells, RUFY3 was found associated in large vesicle structures and to co-immunoprecipitate with Rab5, via an interaction with its carboxyl terminal domain and surprisingly not its RUN domain (Yoshida et al., 2010). Like RUFY2, RUFY3 was also shown in a 2-hybrid screen and by co-immunoprecipitation to bind Rab33, through its coiled-coil domain 1 (CC1; Fukuda et al., 2011). In 293T and 3Y1 cell lines however, RUFY3 was shown not to interact with several small GTPases, including Rab2, Rab5, Rab7, Rho, and Ras. This suggests that either RUFY3 requires cell specific partner proteins or post-translation modifications to be able to bind to small GTPases. RUFY3 was first described as interacting with Rap2, a small Ras-like GTPase, via a 173 residue fragment (83–255) located in the RUN domain (Janoueix-Lerosey et al., 1998; Kukimoto-Niino et al., 2006; Table 1). Together with Rap1, Rap2 interacts with Ras effectors, such as Raf, PI3K, and Ral guanine nucleotide dissociation stimulator, inhibiting activation of their downstream targets, and thus suppressing Ras oncogenic activity (Kukimoto-Niino et al., 2006; Nussinov et al., 2020). In the adult nervous system, Rap1 and Rap2 also regulate the maturation and plasticity of dendritic spine and synapses. By forming a complex together with Rap2 and Fascin, RUFY3 interacts with the filamentous actin network and controls the growth of axons and neuronal growth cone (Wei et al., 2014; Table 1). Recent mechanistic studies indicate that RUFY3 accumulates in lipid rafts by forming a Glycoprotein M6A (GPM6a)-RUFY3-Rap2-STEF/Yial2 complex (Honda et al., 2017a; Table 1). This complex activates the Rac guanine nucleotide exchange factor (Honda et al., 2017b), impacting actin organization and promoting neuronal polarity and growth (Figure 4B). RUFY3 seems therefore to have different axogenic functions in brain (Mori et al., 2007; Honda et al., 2017b) and not surprisingly, roles for RUFY3 in amyotrophic lateral sclerosis (Arosio et al., 2016), major depressive disorder (Aberg et al., 2018) and AD (Zelaya et al., 2015) have been reported. Olfactory dysfunction occurs in 90% of AD cases and is correlated with elevated *rufy3* expression in glomerular and mitral layers of the olfactory bulb (Zelaya et al., 2015). RUFY3 is cleaved by caspase 3 and critically required for caspase-mediated degeneration of tropomyosin receptor kinase A positive sensory axons *in vitro* and *in vivo* (Hertz et al., 2019; Figure 4C). Removal of neuronally enriched RUFY3 is able to block caspase 3-dependent apoptosis, while dephosphorylation of RUFY3 at residue S34 appears required for its degradation (Hertz et al., 2019). Analysis of *rufy3*-deficient mice supports a second distinct function for RUFY3 in neuronal growth and polarity, since mutant embryos show defects in axonal projection patterns. These occur in addition to the prevention of CASP3-dependent apoptosis in dorsal root ganglions. RUFY3 appears therefore to be key for nervous system development, remodeling and function, explaining the embryonic lethality displayed upon *rufy3* genetic inactivation in mouse (Hertz et al., 2019).

With the current advance in genomics and single cell RNA sequencing, specific gene expression patterns can be revised and more accurately defined. Analysis of several genomic databases (BioGPS, NCBI, Human Atlas Protein, ImmGen, Ensembl) reveal that, in addition to neurons, RUFY3 expression can be detected in other tissues and cell types. Moreover, the *rufy3* gene appears to have many transcriptional variants, leading to the expression of different protein isoforms. Two of these isoforms display a C-terminal region extended by 150 amino acids, compared to the previously identified neuronal isoform of RUFY3. Importantly, these previously uncharacterized longer isoforms (RUFY3XL) possess the same RUN domain and a putative FYVE domain in their C-terminus (Figure 1A), indicating that RUFY3 is a legitimate member of the RUFY family. In contrast to classic FYVE zinc fingers, genomic databases reveal this putative FYVE domain appears to lack the tandem histidine residue cluster that defines affinity for PtdIns(3)P (Figure 3B). Interestingly, the SH3 binding site embedded in the RUFY1 and RUFY2 FYVE domains is also present in RUFY3XL, suggesting a potential signal transduction activity for this uncharacterized isoform. The translation of *rufy3xl* mRNA into a functional protein and its capacity to bind PtdIns(3)P remain to be demonstrated. If true, a role for RUFY3 in the coordination of endosome dynamics or organelle transport could be hypothesized. This idea is supported by the observation that RUFY3 is present in Staufen2-containing messenger ribonucleoprotein particles, that are used to transport mRNAs along neuronal dendrites to their site of translation (Maher-Laporte et al., 2010). FYVE proteins have already been implicated in endosome-mediated transport of mRNP (Pohlmann et al., 2015) and RUFY3XL could therefore also perform this function. The existence of FYVE domain bearing isoforms, might extend and diversify its function in other specialized cells.

## RUFY4

RUFY4 is a 64 kDa that is atypical among the RUFY family members, since it bears several non-conserved residues in its RUN domain and it lacks the tandem histidine cluster and the SH3 binding domain normally present in the FYVE domain (Figures 1A, 3A,B). RUFY4 was shown to interact with PtdIns(3)P enriched membranes (Terawaki et al., 2015, 2016). Interestingly, EMBL-EBI and SMART genomic databases show that *rufy4* is present only in mammals, suggesting that *rufy4* is the most recently evolved gene in the RUFY family (Figure 2). RUFY4 levels are extremely low in most cells and tissues with the exception of lungs and lymphoid organs. RUFY4 was found to be strongly induced *in vitro* in dendritic cells differentiated from bone marrow progenitors in presence of GM-CSF and IL-4. *In vivo*, its expression was confirmed in alveolar macrophages and in lung dendritic cells isolated from asthmatic mice (Terawaki et al., 2015). RUFY4 interacts with Rab7 through its RUN domain and promotes the generation of large autophagosomes (Terawaki et al., 2015; Figure 4B and Table 1). RUFY4 over-expression induces the degradation of the autophagy effector LC3/ATG8 and triggers clustering of LAMP1-positive late endosomal compartments. These compartments are distinct from large abnormal autophagosome-like structures positive for RUFY4 and Syntaxin-17, a Qa SNARE involved in autophagosome formation and fusion (Figure 4C). RUFY4 was also proposed to interact with PLEKHM1 and the HOPS complex, which are implicated in LE and lysosome dynamics and positioning (Terawaki et al., 2016). RUFY4 seems therefore able to harness the classical autophagy machinery to facilitate autophagosome formation and increase autophagy flux by acting at different biochemical steps (Terawaki et al., 2015). By optimizing effector protein activity and organelle distribution, RUFY4 expression facilitates the elimination of both damaged mitochondria and intracellular bacteria in phagocytes. RUFY4 expression in HeLa cells can prevent replication of *Brucella abortus* (Terawaki et al., 2015) and *Salmonella typhimurium* (Lassen et al., 2016) suggesting that RUFY4 has a key role in anti-bacterial responses in the lung. It also potentially acts to drive immunity though the regulation of endocytosis and autophagy, necessary for the presentation at the cell surface of antigens from intracellular pathogens (Terawaki et al., 2015).

## FYCO1

FYCO1 is a 150 kDa protein bearing a RUN and a FYVE domains. In several databases, *fyco1* was misidentified as *rufy3*, although these two genes are present on completely distinct chromosomes, in human chromosome 3 and 4, respectively. At the sequence level, although it is larger, FYCO1 appears to be a RUFY4 ortholog gene (Figures 3A,B), suggesting that FYCO1 belongs to the RUFY family. We therefore propose that it could be annotated as RUFY5 to fit the family nomenclature. Separating its N-terminal RUN domain from the FYVE zinc finger, FYCO1 has several CC domains, as well as a LC3/ATG8 Interacting Region (LIR) and a Golgi Dynamic (GOLD) domain in its C-terminus (Figure 1A). FYCO1 preferentially interacts with MAP1LC3A/B of the Atg8-familly proteins through its LIR (Olsvik et al., 2015; Cheng et al., 2016). Coiled-coil domains promote FYCO1 dimerization and have been shown to mediate the formation of a complex with Rab7, via a part of the CC located upstream of the FYVE domain (Pankiv et al., 2010; Wang et al., 2011; Table 1). Overexpression of FYCO1 was shown to redistribute LC3- and Rab7-positive structures to the cell periphery in a microtubule-dependent manner (Pankiv et al., 2010). This effect is mediated by the central part of the CC region and suggests a role for FYCO1 in the transport of autophagic vesicles (Figure 4B). The capacity of FYCO1 to interact with Rab7 and LC3A/B on the external surface of autophagosomes, and PtdIns3P enriched membranes through its FYVE domain, is likely to be key to its function as an adaptor protein. Indeed, these interactions allow microtubule plus end-directed transport and protrusion of endocytic organelles, including autophagosomes (Pankiv and Johansen, 2010), LE (Raiborg et al., 2015, 2016), lysosomes (Mrakovic et al., 2012; Hong et al., 2017; Lie and Nixon, 2019), and phagosomes (Ma et al., 2014). Endoplasmic reticulum (ER) and endosomes are connected through contact sites, the numbers of which increase as endosomes mature. The functions of such sites include to control the association of endosomes with the minus-end-directed microtubule motor dynein and to mediate endosome fission. Repeated LE–ER contacts promote microtubule-dependent translocation of LEs to the cell periphery and subsequent fusion with the plasma membrane (Raiborg et al., 2016). Such fusion induces outgrowth of protrusions and neurites in the neuroendocrine cell line PC12, which require the ER-associated protein protrudin on the ER and FYCO1 to interact with LEs and kinesin 1 (Krauß and Haucke, 2015; Table 1). FYCO1 has been described as a novel mediators of invalopodia formation and function of Protrudin-mediated ER–endosome contact sites (Pedersen et al., 2020). Multiple studies highlight the critical function of FYCO1 in autophagy and autophagosome/endosome trafficking (Dionne et al., 2017) with pathological consequences arising when FYCO1 function is altered (Figures 4B,C). Mutations in the *fyco1* gene affect autophagy and cause autosomal-recessive congenital cataracts by altering lens development and transparency in patients (Chen et al., 2011, 2017; Brennan et al., 2012; Costello et al., 2013; Chauss et al., 2014; Frost et al., 2014; Khan et al., 2015; Gunda et al., 2018; Li et al., 2018). Sequencing studies of candidate genes potentially involved in several neuromuscular or neurodegenerative diseases have identified rare variants of autophagy related proteins like VCP and SQSTM1. Among these genes, a missense *fyco1* variant was identified to cause sporadic inclusion body myositis (Güttsches et al., 2017; Rothwell et al., 2017; Britson et al., 2018; Figure 4C). Finally FYCO1 has been implicated in the autophagic clearance of specialized particles or aggregates, like male germ cell-specific RNP ribonucleoprotein granules (Da Ros et al., 2017), post-mitotic bodies (Dionne et al., 2017) or α-synuclein aggregates (Saridaki et al., 2018).

### RUFY Proteins and Cancer

As describe above, RUFY proteins play a central role in cellular functions by regulating vesicular trafficking and its interactions with the cytoskeleton. Neuronal deficit and neurodegeneration are the most obvious manifestations of RUFY proteins alteration. Not surprisingly, however, given their relatively broad adaptors functions, RUFY proteins have taken center stage in the oncology field.

The ETK tyrosine kinase has been shown to play a pivotal role in a variety of cellular processes including proliferation, differentiation, motility, and apoptosis (Yang et al., 2002; Kung, 2011; Zhuang et al., 2014; Wang et al., 2018). Tyrosine phosphorylation of RUFY1 by ETK appears to be important for its endosomal localization and could play an important role promoting tumoral transformation by affecting downstream effectors of PI3-kinase. RUFY1 was also shown to interact with podocalyxin-like protein (PODXL), a transmembrane glycoprotein with anti-adhesive properties associated with poor prognosis of several cancers (Taniuchi et al., 2018; He et al., 2020; Table 1). Gastric cancer progression is significantly increased upon PODXL expression, a phenotype reduced by concomitant RUFY1 silencing. Depletion of RUFY1 inactivates the PI3K/AKT, NF-κB and MAPK/ERK signaling pathways and reduces drastically migration and invasion of cancer cells *in vitro* (Zhi et al., 2019). Given the positive correlation between *podxl* and *rufy1* expression in tissues and serum, *rufy1* was proposed as a potential biomarker for gastric cancers stratification (Zhi et al., 2019; Figure 3C). Like RUFY1, a role for RUFY2 in various cancer has been reported (Shin et al., 2011; Zheng et al., 2014; Staubitz et al., 2019). *Rufy2* is one of the most frequently mutated genes in high-microsatellite instability tumors and colorectal cancer (Shin et al., 2011). Gene rearrangement of the proto-oncogene *ret* with *rufy2* have been shown to drive tumorigenesis in lung adenocarcinoma (Zheng et al., 2014) and papillary thyroid carcinoma (Staubitz et al., 2019). The gene rearrangement leads to a fusion of the RET tyrosine kinase domain with RUFY2 RUN domain and coiled-coil domain; this appears to be critical for tumorigenesis (Staubitz et al., 2019; Table 1). *Rufy2* mRNA is the target of several microRNAs, including miR-146a, miR-196a-5p and miR-155 (Bofill-De Ros et al., 2015). Dysregulated microRNA targeting of RUFY2 expression was found important for the development of human glioblastoma and ovarian cancer, suggesting a tumor suppression role for RUFY2 (Lukács et al., 2019; Zheng et al., 2020; Figure 4C). Given the key role of RUFY3 in cell migration, membrane transport, and cellular signaling, through its interaction with rap2, it is not surprising that RUFY3 dysregulation has been implicated in several cancer processes and metastatic tumor spread. The abnormal expression of RUFY3 is linked to poor prognosis. It can promote growth, invasion and metastasis in lung adenocarcinoma, gastric cancer cells or colorectal cancer (Xie et al., 2017a, b; Men et al., 2019; Zhu et al., 2019). RUFY3 overexpression and its interaction with P21-activated kinase-1 (PAK1) leads to the formation of F-actin-enriched protrusive structures, increased epithelial-mesenchymal transition and gastric cancer cell migration (Kumar and Vadlamudi, 2002; Vadlamudi and Kumar, 2003). Several transcription factors, including Forkhead box k1 (FOXK1) and Homebox D9 (HOXD9) involved in cancer progression (Moens and Selleri, 2006; Tabuse et al., 2011; Lv et al., 2015; Peng et al., 2016; Wu et al., 2016; Liu et al., 2018; Zhu et al., 2019), have been shown to regulate RUFY3 expression and activity (Xie et al., 2017a; Zhu et al., 2019; Table 1). So far, no correlation has been found between RUFY4 and any type of cancer. FYCO1 has also been implicated in colorectal cancer progression (Sillars-Hardebol et al., 2010) and recent studies have concluded that FYCO1 may serve as a biomarker in bladder cancer (Eissa et al., 2017) or hepatocellular carcinoma (Vongchan and Linhardt, 2017; Figure 4C). Plus, FYCO1 can indirectly associated with cell invasion (Pedersen et al., 2020).

## Conclusion

Although they have been poorly characterized to date, RUFY proteins play a central role in cellular homeostasis by regulating endocytosis, autophagy and coordinating organelle transport with signal transduction cascades. It is important to note that RUFY proteins also provide a regulatory link between cytoskeletal dynamics and membrane trafficking. Consequently, these proteins have adaptive functions by acting on localized actions (through PtdIns(3)P) and signaling (through small GTPases), which can affect key biological functions in specialized cells, such as migration, tissue repair or targeted secretion. The dysregulated expression of RUFY proteins has therefore severe consequences on cell differentiation and polarization, causing cancers or neurodegenerative diseases. However, further molecular and physiological analyses will be required to understand how these proteins exert their functions in specialized cell types like immune cells or neurons. Immunocytes require endocytosis and migration to perform their functions within primary and secondary lymphoid organs or at sites of infection. The restricted expression of RUFY4, as well as the existence of splicing variants of RUFY3 in alveolar macrophages and dendritic cells, suggest a role for these molecules in phagocytes. Of importance will be the characterization of the different molecules interacting either with their RUN or FYVE domains in a cell specific manner. Identification of these RUFY’s interactors will be crucial to establish the functionality of the domains and their importance for signaling on one end and subcellular targeting at the other end. The coiled-coil structural domains found in the central part of the RUFY proteins should also be scrutinized. CC domains, in addition to support homodimerization and increase affinity for PtdIns(3)P, could also be determinant in promoting RUFY proteins interactions with effector molecules, like Rab7, as observed for FYCO1. The nature and pattern of expression of these effector molecules will allow to sort the different activities displayed by the RUFYs in individual cell types and thereby shed light on their physiological importance in health and diseases.

## Author Contributions

Both authors contributed equally to the design and implementation of the research and writing of the manuscript.

## Conflict of Interest

The authors declare that the research was conducted in the absence of any commercial or financial relationships that could be construed as a potential conflict of interest.
